# On-chip Raman spectroscopy of live single cells for the staging of oesophageal adenocarcinoma progression

**DOI:** 10.1038/s41598-024-52079-3

**Published:** 2024-01-19

**Authors:** Alisha Farooq, Christopher D. Wood, John E. Ladbury, Stephen D. Evans

**Affiliations:** 1 School of Physics and Astronomy, University of Leeds, Leeds, UK; 2 School of Molecular and Cellular Biology, University of Leeds, Leeds, UK; 3 School of Electronic and Electrical Engineering, University of Leeds, Leeds, UK

**Keywords:** Biological techniques, Biophysics, Cancer, Cell biology, Oncology

## Abstract

The absence of early diagnosis contributes to oesophageal cancer being the sixth most common cause of global cancer-associated deaths, with a 5-year survival rate of < 20%. Barrett’s oesophagus is the main pre-cancerous condition to adenocarcinoma development, characterised by the morphological transition of oesophageal squamous epithelium to metaplastic columnar epithelium. Early tracking and treatment of oesophageal adenocarcinoma could dramatically improve with diagnosis and monitoring of patients with Barrett’s Oesophagus. Current diagnostic methods involve invasive techniques such as endoscopies and, with only a few identified biomarkers of disease progression, the detection of oesophageal adenocarcinoma is costly and challenging. In this work, single-cell Raman spectroscopy was combined with microfluidic techniques to characterise the development of oesophageal adenocarcinoma through the progression of healthy epithelial, Barrett’s oesophagus and oesophageal adenocarcinoma cell lines. Principal component analysis and linear discriminant analysis were used to classify the different stages of cancer progression. with the ability to differentiate between healthy and cancerous cells with an accuracy of 97%. Whilst the approach could also separate the dysplastic stages from healthy or cancer with high accuracy—the intra-class separation was approximately 68%. Overall, these results highlight the potential for rapid and reliable diagnostic/prognostic screening of Barrett’s Oesophagus patients.

## Introduction

Cases of benign oesophageal diseases such as gastro-oesophageal reflux disease (GORD), eosinophilic oesophagitis, and Barrett’s oesophagus are increasing in prevalence, with the latter classified as the main pre-cancerous condition for oesophageal adenocarcinoma (OAC)^1^. Characterised by the replacement of squamous oesophageal epithelia by a mosaic of intestinal and gastric cell types, Barrett’s Oesophagus is an archetypal metaplastic condition found in up to 10% of GORD patients^2^. Barrett’s Oesophagus occurs as an adaptive response to recurrent acid and bile exposure of the epithelial squamous mucosa, leading to DNA damage, with 0.12–0.6% of patients annually developing low-grade and high-grade dysplasia leading to invasive OAC^3^. Risk factors such as obesity and acid reflux can have a synergistic effect due to increased intra-abdominal pressure and obesity-related metabolic syndrome. Other contributing factors include being of the male sex (7:1 to females), low vegetable and fruit intake, and high red meat, alcohol and tobacco intake^2^. Early detection of dysplastic changes within Barrett’s Oesophagus would allow intervention prior to cancer development. Currently, there is no treatment leading to complete recovery once a patient has been diagnosed.

The integration of multiple disciplines, such as pathology, physiology, and analytical chemistry, has led to the increasing use of microfluidic platforms in cancer research over the last three decades^4,5^. The customisable nature of microfluidic chips has allowed the development of platforms for studying systems from single-cell to patient-derived tissues^6^. The high-throughput, low cost and improved efficiency of these devices provide the ideal platform for identification and characterisation of cancer cell types and metastatic patterns^7,8^. Microfluidic chips have been utilised with other methods of recent cancer research in studies including: tumour cell detection in blood^9,10^, angiogenesis within 3D culture^11^, metastasis of breast cancer^12,13^, and biochemical analysis using Raman spectroscopy, with the latter combination becoming increasingly used^14–17^.

Raman spectroscopy has been previously used for the detection and diagnosis of cancers^18–20^, including to characterise cells during surgical endoscopy^21,22^. Confocal Raman spectroscopy offers diffraction-limited spatial resolution (< 1 μm), which allows micro-spectroscopic analysis of femtolitre volumes within single cells^23^. For Raman spectroscopy to be utilised for single-cell microfluidic clinical screening, an essential requirement is the ability to trap individual cells from a suspension within a flow system^24^. An emerging method for identifying markers across oesophageal tissues^1,25–27^, Raman spectroscopy has potential use as a diagnostic tool for the evaluation of both cancerous and pre-cancerous lesions both in and ex vivo. This includes important studies identifying spectral features associated with different stages of oesophageal adenocarcinoma development using both patient tissues and biofluids^25,26^.

Whilst previous publications detail the use of Raman probes to characterise cells during surgical endoscopy^21,22^, there are no reports characterising the biochemical progression between each stage of Barrett’s dysplasia at a single cell level. Herein, we report live single-cell spectral analysis of six representative cell lines ranging from a healthy oesophageal epithelium (HET-1A) model to oesophageal adenocarcinoma stage III gastric cardia cells (OE19). The four progressive phases of Barrett’s oesophagus, between the healthy epithelium (HET-1A) and the adenocarcinoma (OE19), are represented by cell lines characteristic of low-grade dysplasia (CP-A), mild grade dysplasia (CP-B), moderate grade dysplasia (CP-C) and severe grade dysplasia (CP-D). The microfluidic platform was designed to trap single cells, allowing time-resolved Raman studies to be performed. Principal component analysis (PCA) and linear discriminant analysis (LDA) were used to identify spectral differences associated with progression throughout each stage of diease development with average classification accuracies for all results over 75%.

## Results

### Single cell trapping

**Figure 1:**
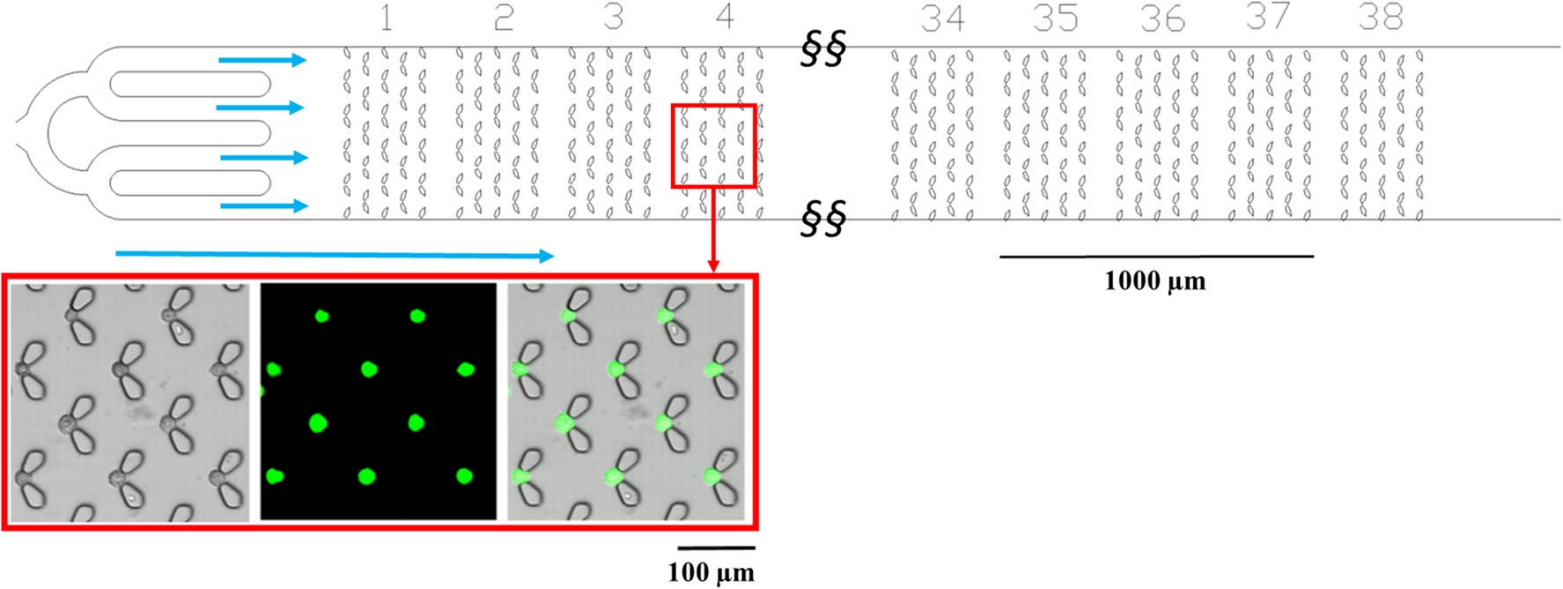
Top: Schematic design of the microfluidic cell traps device. Fluid flow direction is indicated by the blue arrows. Scale bar 1000 µm. Bottom: example of CP-A cells trapped in the device and stained using Calcein AM (live) and Sytox Red (dead) to determine cell viability over 5 h. Bottom left: bright-field image, bottom middle: fluorescence and bottom right: bright field and fluorescence overlay.

Cells were trapped in the microfluidic device at a flow rate of 3 µL/min, and subsequently maintained at 37.5 °C, with a constant flow of fresh culture media (1 µL/min) for the duration of the experiment. The polydimethylsiloxane (PDMS) chip consisted of an inlet, an outlet, and a chamber containing ~ 1200 cell traps. The traps were designed to minimise the cell-PDMS interaction and PDMS contributions to the Raman spectra. Post-Raman spectral acquisition, the on-chip cell viability was monitored over five hours using a Calcein AM live stain and a Sytox Red dead stain. Figure 1 shows an overview of the chip and trap design, together with bright-field and fluorescence (Calcein AM) images of trapped CP-A cells. Previously reported single-cell hydrodynamic traps have exhibited trapping efficiency between 50 and 80% for different design variations^28,29^. The trap design used here (Fig. 1) exhibited a trapping efficiency of 74% (± 6%), averaged over more than 15 independent experiments.

### Changes with disease development

**Figure 2:**
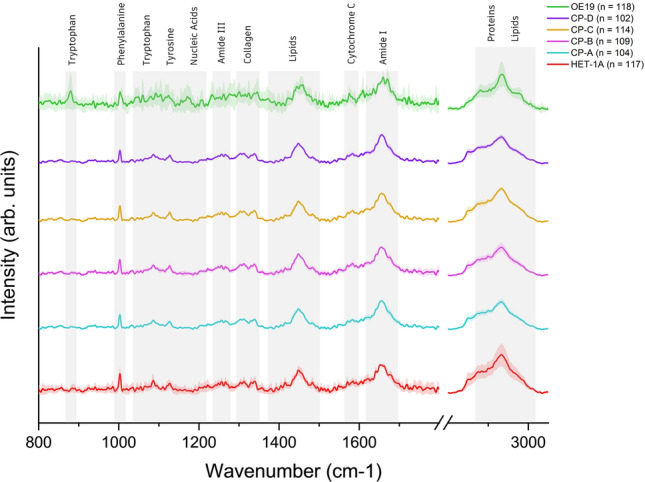
Single-cell Raman spectra representing the progression from healthy squamous oesophageal epithelia (HET-1A: red) through non-dysplastic Barrett’s (CP-A: cyan), mild dysplastic Barrett’s (CP-B: pink), moderate dysplastic Barrett’s (CP-C: orange) and severe dysplastic Barrett’s (CP-D: purple), to oesophageal adenocarcinoma (OE19: green) cells. All spectra were normalized to the Amide I band at 1656 cm^−1^. The shaded area around each cell line is representative of the standard deviation. The grey shading across all cell lines highlights bands of interest (labelled). n = number of single cell Raman spectra. The high wavenumber region (CH_2_CH_3_) has been multiplied by a factor of 0.25 to fit on the same scale.

Raman spectra were obtained from trapped live single cells across the cancerous cell line (OE19), those representing the healthy epithelium (HET-1A) and the intermediary Barrett’s oesophagus (CP-A to CP-D). Figure 2 shows the averaged spectrum and standard error for each cell line, averaging over 100 single cells per sample type. All bands were preprocessed using EMSC and normalised to the Amide I peak at 1656 cm^−1^. Increased aromatic amino acids (tryptophan^30,31^, tyrosine^23,32–34^ and phenylalainine^32,34^) have been associated with cancers such as breast^35^ and colorectal^36^, as well as mutations associated with the acquisition of transforming functions in cancer development^37^. Additional bands related to increased aromatic acids are present within the OE19 cell line, such as the tryptophan shoulder at 1009 cm^−1^^35,37^.

Nucleic acid and protein-related bands, such as Amide III and Amide I, are labelled and present across all six cell lines. DNA-associated bands, as seen at 1089 cm^−1^, are common within transformed cancerous cells^34^, with an increased DNA concentration associated with disease progression. An increase in protein signature is observed via the increase in the CH_3_ stretching (2881 cm^−1^ and 2980 cm^−1^) for the OE19 (cancer) cells. Such an increase in protein content is consistent with previously reported data^25,38,39^.

The CH_3_ and CH_2_ twisting modes previously attributed to collagen^40^ were observed between 1310 and 1340 cm^−1^^37,42^. Lipid-associated bands are reported as being attributed to the acyl (C–C) backbone in lipids^41,42^, CH_3_ rocking/C–O vibrations^26^, bending modes of C–H in CH_2_ moieties^43,44^ as well as methylene deformation^34^. These changes in cellular lipid quantity are significant biomarkers of abnormal membrane composition, characteristic of neoplastic cells^45^.

### Comparison of healthy versus cancer cells

**Figure 3:**
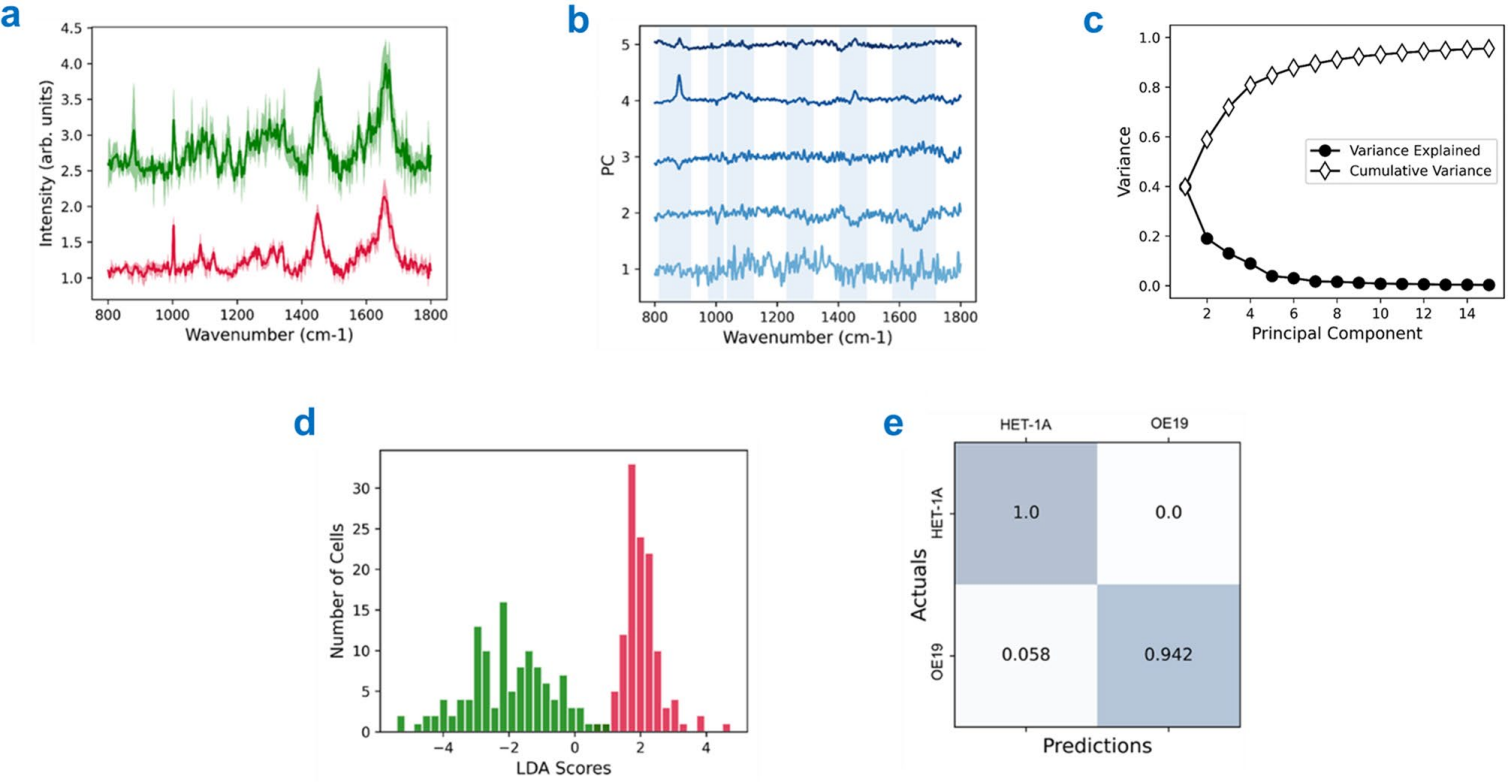
PCA-LDA of HET-1A (red) and OE19 (green) EMSC-corrected spectral data. (**a**) Truncated Raman datasets between 800 and 1800 cm^−1^ used for analysis. (**b**) Principal component loadings taken from PCA. Scores 1–5 are shown, accounting for > 85% variability. Bands of interest are highlighted in blue. (**c**) Cumulative variance and variance explained for the first 15 PCs. (**d**) LDA histogram displaying the spread of the scores for each dataset. (**e**) Confusion matrix for statistical analysis of the PCA-LDA. The outputted values are representative of the False Positive (0.058), True Positive (1), False Negative (0.942) and True Negative (0).

Analysis was carried out to determine if the OE19 oesophageal adenocarcinoma cell line could be distinguished from HET-1A (healthy) cells. PCA was conducted as an unsupervised technique with the goal of maximising and identifiying the variance of the data along the principal components (PCs). These PCs can be visualised and plotted to identify features within the spectra that help distinguish the different cell lines. PCA was undertaken using the first 15 PCs of a truncated Raman spectra (Fig. 3a). Differences across the first 5 PCs, accounting for > 80% variance, are shown in Fig. 3b, with bands showing significant differences highlighted in blue. The cumulative variance and variance explained for all 15 PCs is shown in Fig. 3c. Variation within the amino acid bands (tryptophan, tyrosine and cytosine) are seen in the first 5 PCs at 880 cm^−1^, 1171 cm^−1^ and 1610 cm^−1^ respectively. Additional bands showing variation across all 5 PCs include DNA (1089 cm^−1^), collagen (1310–1340 cm^−1^), methylene deformation (1450 cm^−1^) and Amide I (1656 cm^−1^).

Further analysis was undertaken using LDA, a supervised technique used for classification, by maximising the separation between different classes within a dataset via their discriminant functions. The LDA was trained on 75% of the data and tested on the remaining 25% using a fourfold cross-validation approach. The combined PCA-LDA was able to separate the datasets into distinct classes with high accuracy. The histogram (Fig. 3d) indicates a clear separation of both populations. It is also evident that the representative healthy cell line (HET-1A, red) provides a narrower distribution compared to the cancerous equivalent (OE19, green).

The confusion matrix obtained from this analysis is shown in Fig. 3e. Whilst all HET-1A cells were correctly categorised, approximately 6% of OE19 cells were mistaken for HET-1A, this is presumably a consequence of their heterogeneity. The precision, recall and F1-score (defined in Supplementary Fig. S1) were determined to be 1, 0.972 and 0.942 respectively. The average accuracy for the classification of healthy vs. cancer single cells was 97%. 

### Analysis of Barrett’s oesophagus cell lines

**Figure 4:**
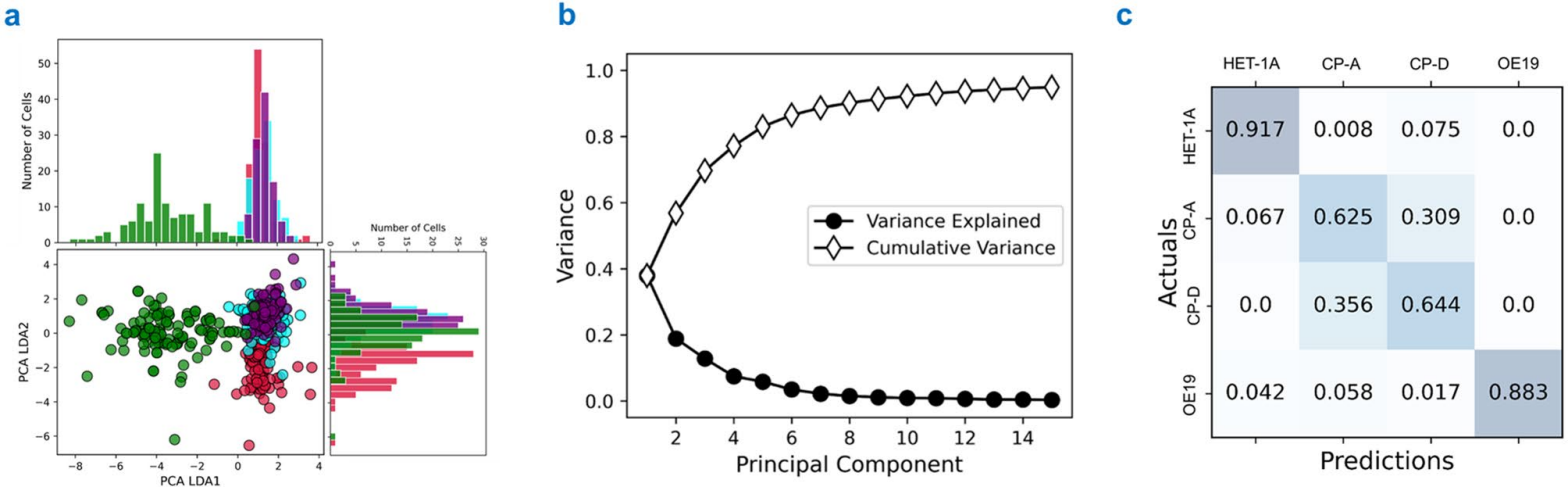
(**a**) 2D plotted PCA LDA1 vs PCA LDA2 scores for HET-1A (red), CP-A (cyan), CP-D (purple) and OE19 (green) and their relative LDA histograms. (**b**) Cumulative variance and variance explained for the first 15 PCs. (**c**) Confusion matrix for statistical analysis of the PCA-LDA.

Intermediate between the healthy (HET-1A) and cancerous (OE19) stages are the progressive stages of Barrett’s oesophagus, represented by the cell lines CP-A (non-dysplastic) and CP-D (severe dysplastia). Shown in Supplementary Fig. S2, the spectra for each cell line was truncated to 800–1800 cm^−1^ prior to PCA-LDA. Analysis of the first 5 PC loadings, covering 85% variance (Fig. 4b), confirms the influence of amino acid bands, with phenylalanine and tryptophan bands present across all 5 PCs. Other cellular components such as Amide III, Lipids and Amide are present in PCs 1 and 5, PCs 2, 4 and 5, and PCs 2 and 5, respectively. Additionally, between disease stages, the Cytochrome C (Cyt C) band (1583 cm^−1^) is present within PC1 and PC2. This peak can be attributed to the presence of aromatic side chains within the Cyt C compound^46–48^.

The 2D scatter plot for the LDA is displayed in Fig. 4a, with HET-1A (red), CP-A (cyan), CP-D (purple), and OE19 (green), along with their respective histograms.

From this scatter plot we see that the OE19 dataset is separated from the others and also shows greater scatter as might be expected from a cancerous dataset with uncontrolled mutation. The HET-1A appears to be separated from CP-A and CP-D datasets in LDA2, whilst the CP-A and CP-D cells appear to exhibit strong overlap. This is further supported by looking at the confusion matrix in Fig. 4c, which suggests that neither of the Barrett’s Oesophagus cell lines (CP- A and CP-D) nor the healthy cell line (HET-1A) were mis-categorised as the cancer cell line (OE19). The HET-1A cells were correctly identified with ~ 91% accuracy whilst the non-dysplastic (CP-A) and dysplastic (CP-D) cell lines were only correctly classified correctly 63% and 64% of the time. The largest errors occur between CP-A and CP-D cells, with ~ 36% inaccuracy. The OE19 cells could be inaccurately categorised as any of the other three cell lines but with low probability—HET-1A (4%), CP-A (6%) and CP-D (2%). Supplementary Table S1 provides the precision, recall and F1-scores for these results.

### Dysplastic progression within Barrett’s oesophagus

Next we focused on the analysis of cell lines representing high-grade Barrett’s dysplasia. Identification of measurable differences in the spectral data could provide a promising route to the diagnosis of Barrett’s Oesophagus severity in patients.

**Figure 5:**
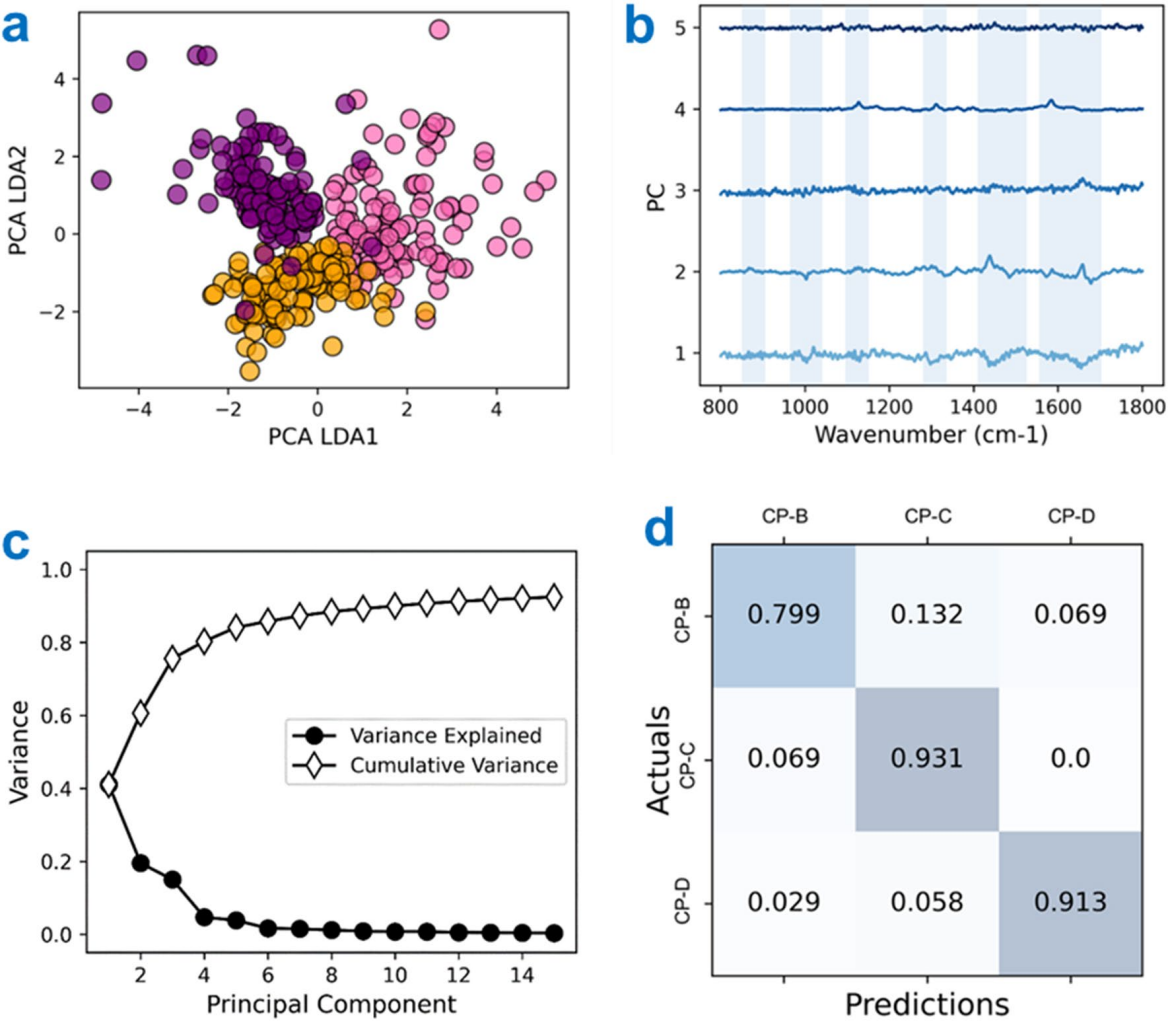
(**a**) 2D plotted PCA LDA1 vs PCA LDA2 scores for CP-B (pink), CP-C (orange) and CP-D (purple). (**b**) Principal component loadings taken from PCA. Scores 1–5 are shown, accounting for > 80% variability. Bands of interest are highlighted in blue. (**c**) Cumulative variance and variance explained for the first 15 PCs. (**d**) Confusion matrix for statistical analysis of the PCA-LDA.

The PCA-LDA was carried out on the fingerprint region of the dysplastic cell lines CP-B (pink), CP-C (orange) and CP-D (purple), shown in Fig. 5. The first 5 PC loadings in Fig. 5b were responsible for 80% of the variance, with the cumulative variance and variance explained detailed in Fig. 5c. Negative bands in PC1 correlated with the aromatic amino acid content and Cyt C. These bands become positive between PC2 and PC4. Other notable bands present in PC1 are associated with Amide I, Amide III, lipid and collagen content. The PO_2_^−^ DNA-associated band (1089 cm^−1^) also displays an increase in PC1. The scatterplot, Fig. 5a., shows that the different cells types are well separated by LDA1 and 2. The confusion matrix for this data is shown in Fig. 5d, with precision, recall, and F1-scores given in Supplementary Table S2. The average accuracy was calculated at 92%, with PCA-LDA classification accuracy at 77%.

The PCA-LDA is able to correctly identify each stage of Barrett’s dysplasia with accurate detection of CP-B at 80%, CP-C at 93% and CP-D at 91%. With CP-B cells have a 13% chance of being identified as CP-C cells.

This low risk of misclassification identifies a new method of detecting progression within Barrett’s dysplasia at a single-cell level, which could allow earlier detection of progression towards cancer within a clinical setting. The results from this work not only highlight the use of single-cell Raman spectroscopy alongside PCA-LDA for the identification of healthy versus cancer cells but also for distinguishing between progressive disease stages within Barrett’s Oesophagus dysplasia itself.

## Discussion

Raman spectroscopy provides a “fingerprint” of the molecular covalent bonds present within a biological sample and importantly how they change between different disease states. For example, in this research it is shown how healthy oesophageal cells exhibiting progression of Barrett’s Oesophagus toward adenocarcinoma have distinct spectral signatures. In the case of the OE19 cell line, peaks associated with changes in the DNA backbone geometry (Fig. 5b) are indicative of phosphate ion interactions found in neoplastic cells^32,38,49^, arising due to the erratic accumulation of DNA and RNA in the nuclei of cancer cells^23^.

The increase of the aromatic amino acids associated with advanced disease progression is of note, with previous papers also highlighting the influence of these compounds in various cancers and diseases^35,50^. These compounds have been shown to play a significant role in orchestrating morphological changes within malignant tissues^51^. Moreover, the increase in transcription and replication of cancerous cells requires a concomitantly increased volume of proteins for sustaining the excessive RNA synthesis. This increase in protein expression during cancer progression is expected due to an overall increase in protein overexpression with multiple oncogenic pathway activation and upregulation^25,38,39^, as shown in Fig. 2.

Increase in cellular lipid quantity across cell lines (Fig. 2) are significant biomarkers of abnormal membrane composition, characteristic of neoplastic cells. These increases occur due to the modulation of specific enzymes and pathways within the cells, allowing for a higher proliferation rate, increased chemotherapy resistance and attenuation of the immune response^45^.

The use of microfluidic devices to trap single cells significantly reduces the volume of reagents required during the treatment and staining of cells. Further, the regular array structure permits the facile and reproducible location of the same cells for multiple measurements over long time courses. In principle, the trapping of cells on a regular array also permits automated cell analysis. This approach offers several potential advantages (i) it could remove the need to culture patient samples, as only a small number of cells are required for testing, and (ii) it is compatible with emerging techniques of sample collection^52,53^, which could remove the need for invasive biopsies. The microfluidic platform can be utilised for dissociated cells from patient tissues, or representative cell lines, to allow studies of tumour heterogeneity and/or responses to drug or environmental changes. Our choice of a 60 s acquisition time was a compromise between obtaining a high signal:noise ratio and the time taken to obtain spectra. This could be reduced but would need to be done in conjunction with a study on potential adverse effects on classification accuracy. Alternative approaches, such as Stimulated Raman Spectroscopy, could decrease acquisition times, but the potential compromise associated with the reduction in spectral resolution would need to be considered.

The use of vibrational spectroscopy for quantitative biochemical cellular analysis may provide a non-invasive detection method of disease to allow the classification and grading of dysplasia and malignant neoplasms**.** The ability to differentiate between these stages has the potential to advance clinical observation of Barrett’s Oesophagus progression, potentially allowing earlier intervention and more frequent check-ups for Barrett’s Oesophagus patients who are deemed at high risk for cancer development. Within a clinical and research setting, each stage of Barrett’s Oesophagus dysplasia is often difficult to distinguish from each other. For continuation of this work, patient samples with a known diagnosis would help in verifying this method as a possible diagnostic tool within a clinical setting. These results highlight not only the potential of combining microfluidic devices with Raman spectroscopy allowing for accurate and sensitive biochemical quantification of disease progression, but also the potential to achieve this using single cells.

## Materials and methods

### Cell lines

Six cell lines were acquired for these experiments. Human oesophageal cell line HET-1A (ATCC® CRL-2692™) was used for the healthy control. The Barrett’s oesophagus in vitro model comprised of one non-dysplastic cell line and three high-grade dysplastic cell lines: CP-A/KR-42421 (ATCC® CRL-4027™), CP-B/ CP-52731 (ATCC® CRL-4028™), CP-C/CP-94251 (ATCC® CRL-4029™) and CP-D/CP-18821 (ATCC® CRL-4030™), respectively. The cell line OE19/JROECL19 (ECACC 96071721) was used as a representative for oesophageal adenocarcinoma. All cell lines were authenticated and confirmed negative for mycoplasma infection.

### Cell culture

Healthy (HET-1A) and Barrett’s Oesophagus cell lines (CP-A, CP-B, CP-C and CP-D) were cultured in keratinocyte serum-free media (KSFM, Invitrogen), and the oesophageal adenocarcinoma cell line (OE19) was cultured in RPMI cell media containing 2 mM glutamine (Thermo Fisher, UK). Both cell media were supplemented with 10% foetal bovine serum (Merck Life Science Limited). Cells were washed and passaged using Dulbecco's Phosphate Buffered Saline (Merck Life Science Limited) and TrypLE Express Enzyme (Thermo Fisher, UK). Culture was maintained at conditions of 37 °C and 5% CO_2_ with a maximum passage number of 20. Cells were passaged immediately before experimentation and re-suspended in their appropriate culture medium before flowing through the respective microfluidic device at a flow rate of 3 µL/min.

Cellular viability on-chip was determined by running both a live and dead stain through the device at 3 µL/min after each experiment (Viability/Cytotoxicity Assay Kit, Calcein AM, Sytox Red—L32250, Thermo Fisher, UK). A 488 nm optically pumped semiconductor laser aided sequential excitation and fluorescence emission was measured from 490 to 525 nm. Images were captured using a 10 × dry objective.

### Polydimethylsiloxane (PDMS) microfluidic device fabrication

PDMS (Sylgard, 184, Dowsil) base: curing agent was mixed in a 10:1 ratio before pouring and curing over the designed master wafer. Oxygen plasma bonding (100 W, O2 pressure 0.5 mbar, 30 s, Zepto Plasma Unit, Diener Electronic, Germany) was used to bind each PDMS device to a 50 × 20 mm glass coverslip. Devices were sterilised and washed prior to experimentation by pumping through 70% ethanol and Mill-Q consecutively via the use of a PHD ULTRA advanced syringe pump (Harvard Apparatus, USA) at a flow rate of 100 µL/min.

Measurements were taken using an integrated Raman system consisting of an inVia Raman confocal inverted microscope (Renishaw) and a Leica DMi8/SP8 laser scanning confocal microscope. A 532 nm DPSS diode laser, supplying a laser power of 22 mW, at the sample surface, was used with a grating of 1800 lines mm^−1^. A near-infrared enhanced CCD array detector (1024 × 256 pixels, Renishaw) was used to collect light. The Raman system was calibrated to the Si band position (520.5 cm^−1^) prior to each experiment. To ensure no adverse effect due to irradiation, 12 consecutive spectra were obtained from the same “live” cell, these showed no compromise in cellular viability (morphological changes, or spectral differences).

All single-cell spectral data were obtained with the use of a 100 × oil objective (HC PL APO CS2 FWD 0.13 mm NA 1.4. A beam expander was positioned in the optical path between the excitation laser and the objective lens used to focus on the sample. This was used to defocus the laser spot via the Renishaw WiRE software, with 0% defocus (representing the diffraction-limited spot size of the laser), and 100% defocus producing a maximum spot size (dependent on the objective). A 20% defocus, when used with a 100 × objective, produced a spot size of approximately 18 μm at the sample. The entrance slit was set to 20 μm. Spectral settings remained the same across all six cell lines, utilising a step configuration with a 5 s exposure time and 6 accumulations in two different windows (300–1800 cm^−1^ and 1800–3200 cm^−1^), giving a total exposure time of 60 s per cell. Single Raman spectra was taken from > 100 cells for each cell line. The exact number of single-cell spectra per cell line are as follows: HET-1A (117), CP-A (104), CP-B (109), CP-C (114), CP-D (102), and OE19 (118). Background spectra was acquired at the same Z-position as the cells.

### Raman data pre-processing

Individual spectra were compiled into datasets corresponding to each cell line using Matlab’s Statistics and Machine Learning Toolbox (MathWorks) with the silicon calibration band for each experiment used to calibrate the wavenumber axis of each spectrum. PDMS background contributions were removed from the data using a modified extended multiplicative signal correction (EMSC) technique^52^. An example of EMSC-corrected CP-A spectra is shown in Supplementary Fig. S4. Dataset files were exported to Python where the outliers with the highest variance were removed to give a final dataset of approximately 100 single-cell spectra per cell type.

All spectra was baseline corrected using the algorithm developed by Koch et al.^53^ and truncated to only consider the fingerprint region between 800 and 1800 cm^−1^. To allow for comparison against other biochemical Raman literature, the spectra were normalised to the Amide I band at 1656 cm^−1^. This was done for each cell line. Other pre-processing steps included smoothing [polynomial 2, window 17], averaging and calculation of the standard deviation.

### PCA-LDA single cell analysis

Principal component analysis and linear discriminant analysis (PCA-LDA) was conducted using the EMSC-corrected datasets shown in Fig. 2, truncated within the fingerprint region (800–1800 cm^−1^). All confusion matrices presented from this data are normalised using the fourfold cross-validation method.15 principal components were chosen for the PCA as this was representative of > 90% of the overall variance within the data. The variance explained reaches a plateau after 15 PCs, indicating that the remaining components are representative of little to no change. The fourfold cross-validation was conducted using a StratifiedKFold, whereby the datasets for each set of analysis were split into k (4) groups of equal size, with the first group held as a validation set, and the remaining groups (k − 1) used to fit the method.

### Supplementary Information

Supplementary Information.

## Data Availability

The data presented in this study’s findings will be available at 10.5518/1246.
