# Supplementary material for: On-chip Raman spectroscopy of live single cells for the staging of oesophageal adenocarcinoma progression
Source: Sci Rep. 2024 Jan 19;14:1761. doi: 10.1038/s41598-024-52079-3 (PMC10799027; doi:10.1038/s41598-024-52079-3)
Supplement: Supplementary file 1 — Supplementary Information. [file 41598_2024_52079_MOESM1_ESM.docx]

| Cell line | Precision | Recall | F1-Score |
| --- | --- | --- | --- |
| HET-1A | 0.917 | 0.917 | 0.905 |
| CP-A | 0.624 | 0.624 | 0.610 |
| CP-D | 0.644 | 0.644 | 0.63 |
| OE19 | 0.833 | 0.833 | 0.938 |

Supplementary Table S1: Precision, recall and F1-scores for 4-fold cross-validation analysis of the LDA scores for HET-1A (healthy), CP-A (non-dysplastic), CP-D (severely dysplastic), and OE19 (cancerous) single cells. The average model accuracy for this analysis was 88 %.

| Cell line | Precision | Recall | F1-Score |
| --- | --- | --- | --- |
| CP-B | 0.799 | 0.799 | 0.8424 |
| CP-C | 0.931 | 0.931 | 0.878 |
| CP-D | 0.913 | 0.913 | 0.921 |

Supplementary Table S2: Precision, recall and F1-scores for 4-fold cross-validation analysis of the LDA scores for CP-B (mild dysplasia), CP-C (moderate dysplasia) and CP-D (severe dysplasia) single cells. The average accuracy for this classification model was 92 %.


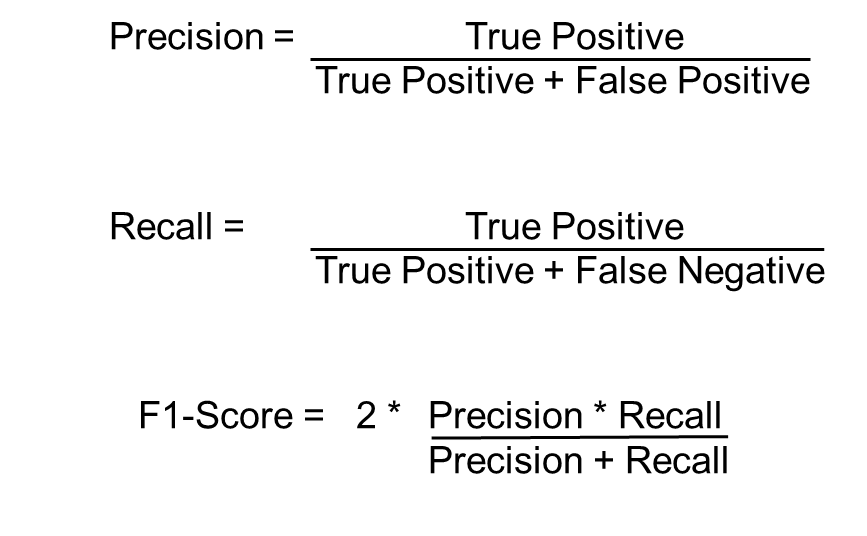


Supplementary Figure S1: Definitions for precision, recall and F1 score as used for statistical analysis. Precision, or positive predictive value, can be calculated by dividing the true positive value over the sum of the true positive + the false positive. Recall, also known as sensitivity or true positive rate, is determined by dividing the true positive value over the sum of the true positive + false negative. The F1 score is accounts for both the precision and the recall value, calculating the mean and gives us a better measure than accuracy alone. For this, divide the sum of (precision * recall) over (precision + recall) and multiplying the output value by 2. The F1 score is a larger value only if the precision and recall values are close to 1.


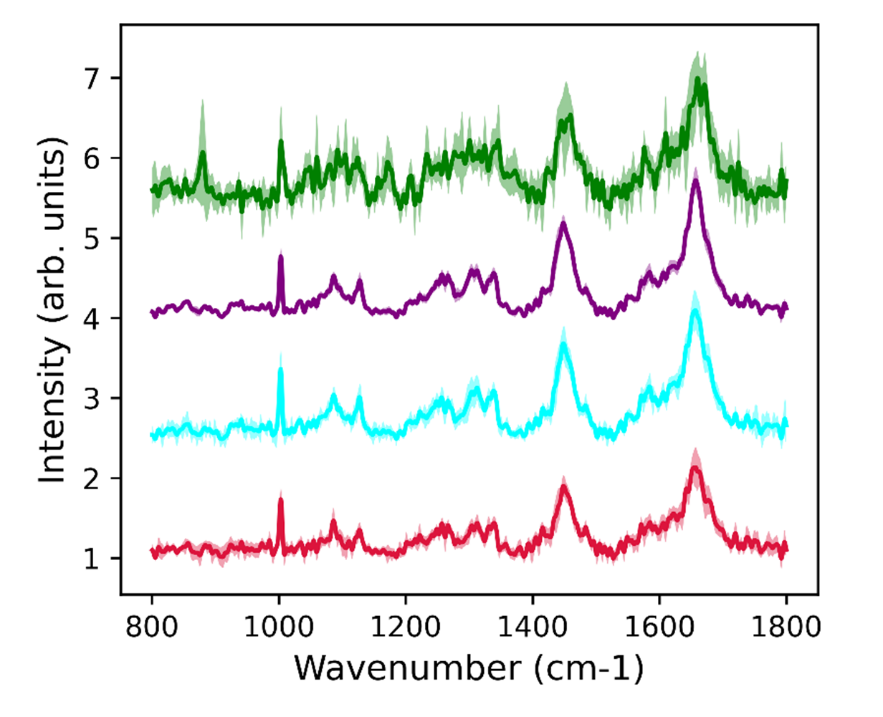
Supplementary Figure S2: Truncated Raman spectra (800 -1800 cm-1) for each stage of cancer development. The healthy (HET-1A), non-dysplastic (CP-A), severely dysplastic (CP-D) and cancerous (OE19) cell lines are represented by red, cyan, purple and green spectra, respectively*.*


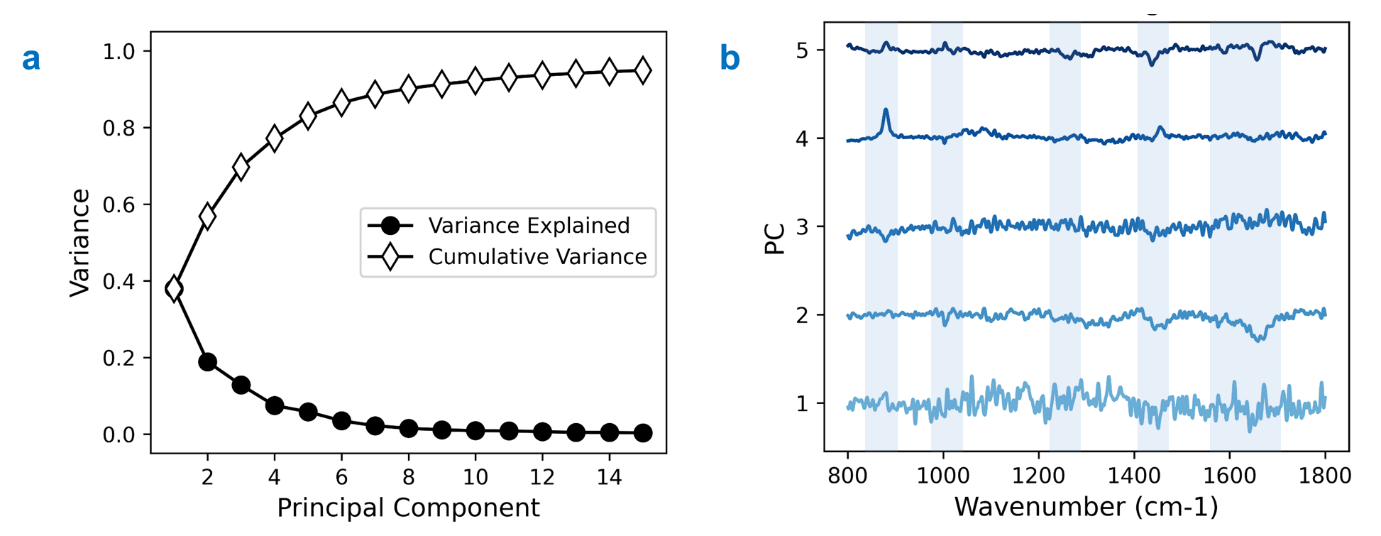


Supplementary Figure S3: (a) 2D plot representing the variance explained and the cumulative variance for HET-1A (healthy), CP-A (non-dysplastic), CP-D (severely dysplastic) and OE19 (cancerous) single cells. (b) The PC loadings from the data shown in Figure 4. Bands of interest have been highlighted in blue.

.


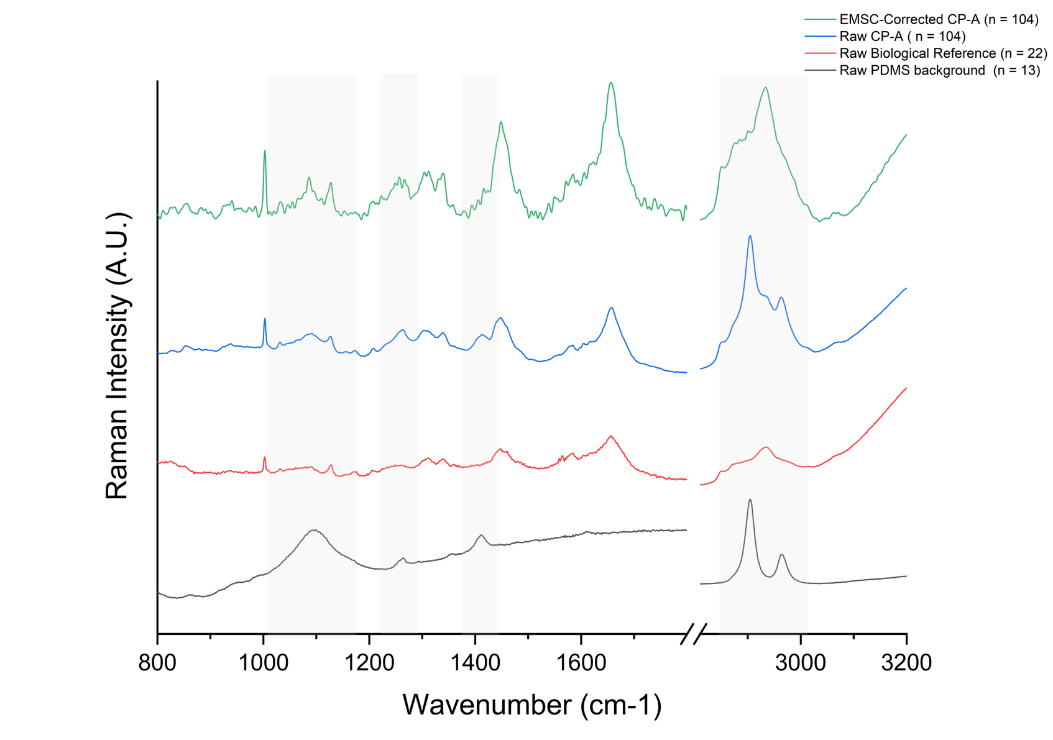
Supplementary Figure S4: Raman spectral plot demonstrating extended multiplicative signal correction (EMSC) on non-dysplastic (CP-A) single cell data. Offset spectrum of PDMS (grey), biological CP-A single cells on quartz (red) and raw CP-A single cells (blue) is shown in ascending order, with the corrected spectrum (green). Grey shaded bands are shown to highlight the removal of the PDMS bands in the final spectrum used for analysis.

*.*
